# Surgical Interventions in Advanced Hidradenitis Suppurativa: A Systematic Review

**DOI:** 10.1177/12034754251391811

**Published:** 2025-11-12

**Authors:** Michal Moshkovich, Edgar Akuffo-Addo, Emily Volfson, Jonah Perlmutter, Rebecca Yakubov, Romy Levy, Amrit Thandi, Ammar Saed Aldien, Katya Peri, Ilya Mukovozov

**Affiliations:** 1 Temerty Faculty of Medicine, University of Toronto, Toronto, Ontario, Canada; 2 Division of Dermatology, Department of Medicine, University of Toronto, Toronto, Ontario, Canada; 3 Faculty of Medicine, University of Ottawa, Ottawa, Canada; 4 Faculty of Medicine and Health Sciences, McGill University, Montréal, Québec, Canada; 5 Department of Medicine, Division of Dermatology, Women’s College Hospital, Toronto, Ontario, Canada

**Keywords:** Hidradenitis suppurativa, wide excision, graft, flap, laser

## Abstract

**Background:**

Hidradenitis suppurativa (HS) is a chronic, immune-mediated skin disorder affecting intertriginous areas, frequently leading to painful nodules, abscesses, sinus tracts, and scarring. In patients with moderate-to-severe disease (Hurley Stages II and III), surgical intervention is frequently required to achieve durable disease control.

**Objective:**

To compare and evaluate the surgical interventions employed in Hurley Stage II and III HS by evaluating recurrence rates, postoperative complications, and patient-centred outcomes across different operative modalities.

**Methods:**

A comprehensive search of MEDLINE and EMBASE identified studies reporting surgical outcomes in Hurley Stage II and III HS. English-language studies describing postoperative recurrence, complications, and patient-centred outcomes stratified by surgical intervention were included. Of 647 studies screened, 136 studies were included.

**Results:**

A total of 136 studies were included (5,646 procedures). Primary closure had the highest recurrence (38.0%) and complication rates (29.4%). Wide excision (n = 1923) showed moderate recurrence (17.2%) and the highest cosmetic dissatisfaction. Laser-assisted surgery had the lowest complication rate (2.2%) and recurrence rate (5.7%). Flaps and grafts showed higher complication rates but fewer recurrences than primary closure.

**Conclusion:**

Surgical outcomes in advanced HS vary by intervention. Primary closure is associated with the highest rates of recurrence and complications, while wide excision and laser-assisted surgery may offer improved disease control. These findings support individualized surgical planning in Stage II and III HS.

## Introduction

Hidradenitis suppurativa (HS) is an immune-mediated, inflammatory skin condition characterized by painful nodules, abscesses, and draining sinus tracts in intertriginous areas such as the axillae, groin, and inframammary regions.^1^ Its chronic, relapsing course often results in physical discomfort, malodorous drainage, and psychological distress.^2^ HS severity is commonly stratified using the Hurley staging system: Stage I involves isolated abscesses, stage II includes recurrent lesions with tract formation and scarring, and stage III is defined by diffuse involvement with tunnelling networks and extensive inflammation.^3^ Medical therapies, including antibiotics, hormonal agents, and biologics may control early-stage disease, but are often insufficient for Hurley Stage II and III HS.^4-6^ In such cases, surgery is essential to remove irreversibly damaged tissue and reduce the risk of future flare-ups.^7^

A variety of surgical interventions have been described in the literature, ranging from wide excision with healing by secondary intention to primary closure or reconstructive closure using grafts or flaps.^7,8^ Each technique carries risks and benefits, with recurrence rates ranging from 2% to 40%.^9,10^ Complications such as delayed healing, infection, wound dehiscence, and scarring may affect quality of life and long-term satisfaction.^8^ Given the growing number of surgical options for advanced HS and the heterogeneity of outcome reporting across studies, there remains a need for comparative synthesis of complication and recurrence rates associated with each intervention. This systematic review evaluates the therapeutic efficacy of surgical treatments in Hurley Stage II and III HS by analyzing recurrence rates, postoperative complications, and patient-centred outcomes.

## Methods

### Data Sources and Search Strategy

This systematic review was conducted according to the Preferred Reporting Items for Systematic Reviews and Meta-analyses (PRISMA) guidelines. A literature search was conducted using Ovid MEDLINE and Ovid EMBASE to identify studies evaluating surgical interventions for Hurley Stage II and III HS (Supplementary Table 1). The search included English-language studies published from 1947 to October 17, 2024. All references were imported into Covidence for screening and full-text review. Three reviewers independently screened 647 studies, of which 230 were selected for full-text review. Disagreements were resolved by a third reviewer. Following full-text screening, 136 studies met inclusion criteria (Supplementary Table 2).

### Inclusion Criteria

Eligible studies were primary, peer-reviewed articles involving human participants with Hurley Stage II or III HS who underwent surgical treatment. Study designs included randomized controlled trials, cohort studies, case-control studies, case series, and case reports. Inclusion required reporting on at least one outcome related to surgical intervention (eg, recurrence rate, postoperative complications, wound healing). Only full-text English-language studies were considered. Studies focused on Hurley Stage I disease, nonsurgical treatment, and non-human subjects were excluded. Editorials, expert opinions, commentaries, and incomplete conference abstracts were also excluded.

### Data Extraction

Six reviewers extracted data using a standardized chart, collecting study characteristics, patient demographics, surgical details, and outcomes. Primary outcomes were recurrence and complication patterns across interventions, while secondary outcomes included reoperation, cosmetic dissatisfaction, and range of motion (ROM) limits. Data extraction was conducted in 2 phases to ensure accuracy and completeness.

## Outcomes

### Primary Outcomes

Primary outcomes included postoperative disease recurrence and complications stratified by intervention. Recurrence was defined as clinically active lesions reappearing at the surgical site, adjacent areas, or distant sites, during the follow-up period. Reoperations for recurrence were also recorded. Complications included the following: surgical site infection (SSI), wound dehiscence, hematoma, seroma, delayed wound healing (at donor or recipient sites), and graft or flap loss. While the overall complication rate encompassed these events; it is not limited to them, as some studies did not specify the type of complications. Linear and hypertrophic scarring was recorded as a healing outcome.

### Secondary Outcomes

Secondary outcomes included cosmetic dissatisfaction and postoperative ROM limitation. Additionally, the use of negative pressure wound therapy (NPWT) or vacuum-assisted closure (VAC), mean hospital stay duration, recovery time, and follow-up length were assessed as secondary indicators of postoperative burden and health care utilization.

## Results

### Wide Excision

A total of 1923 wide excision procedures were included in this study. Of these, 135 procedures involved the use of NPWT or VAC postoperatively. Cosmetic dissatisfaction was reported in 90 procedures (4.7%), though no cases of linear or hypertrophic scarring were documented. Restricted ROM was noted following 24 procedures (1.2%). Disease recurrence occurred after 331 procedures (17.2%) during the follow-up period. Of these, 24 cases required surgeries for the disease recurrence. Postoperative complications were observed in 140 procedures (7.3%), with the most common being SSIs (n = 19), followed by wound dehiscence (n = 12), delayed wound healing (n = 8), and hematoma (n = 1). No seromas were reported. Reoperation was required after 29 procedures (1.5%). The average length of hospital stay was 7.3 days, and recovery time following surgery averaged 2.8 months. Patients were followed for a mean of 36.2 months, with recurrence typically occurring around 7.3 months postoperatively.

### Sequential Excision

A total of 32 sequential partial excisions were included in the study. None of the patients in this group received NPWT or VAC. There were no reports of dissatisfaction with cosmetic outcomes or postoperative-restricted ROM. Disease recurrence occurred in 9 procedures (28.1%), but none required surgical intervention for recurrence. Only one postoperative complication was reported, which was a case of wound dehiscence. Additionally, no patients required reoperation. Although hospital stay data were not available, average recovery time was 2.0 months. Mean follow-up was 20.4 months, but the timing of recurrence was not consistently reported across studies.

### Laser Surgery

A total of 453 laser surgeries were included in the study with 333 (73.5%) being carbon dioxide (CO2) lasers. Twelve patients received NPWT/VAC. Only one patient (0.2%) reported dissatisfaction with their cosmetic outcome, and no cases of restricted ROM were observed. Disease recurrence was noted in 26 procedures (5.7%) with one case requiring surgery. Thirteen patients developed linear scars and while 39 had resultant hypertrophic scars. Ten postoperative complications (2.2%) were documented: Wound dehiscence occurred in 2 patients, SSIs in 3, and there were no reported cases of hematoma, seroma, or delayed wound healing. Three patients (0.7%) required reoperation. On average, hospital stays lasted 0.5 days, with recovery periods of 2.3 months. Patients were followed for a mean of 35.2 months, and recurrence typically occurred around 6.5 months post-surgery.

### Primary Closure

A total of 1,112 primary closure procedures were included in the study. Of these, 15 patients received NPWT or VAC. Restricted ROM was reported in 30 patients (2.7%), and 42 patients (3.8%) were dissatisfied with their cosmetic outcomes. Disease recurrence occurred in 423 primary closures (38.0%), with 124 patients requiring surgery to manage the recurrence. Scarring was observed in 11 patients, including 9 with linear scars and 2 with hypertrophic scars. There were 327 reported postoperative complications (29.4%), the most common being wound dehiscence (n = 120), followed by delayed wound healing (n = 66), SSI (n = 41), seroma (n = 5), and hematoma (n = 2). A total of 107 patients (9.6%) required reoperation. Mean hospital stay was 4.7 days, and average recovery time was 2.0 months. Follow-up duration extended to 37.9 months, with a mean time to recurrence of 9.3 months.

### Skin Grafts

A total of 933 skin graft procedures were included in the study, of which 715 were split-thickness skin grafts (STSG). Of these, 218 (26.2%) patients had NPWT/VAC. Restricted ROM was reported in 23 patients (2.5%), and 27 patients (2.9%) were dissatisfied with their cosmetic outcome. Disease recurrence occurred in 84 grafts (9.0%). Among these, 39 procedures were performed to address the recurrence. Scarring was reported in 3 patients, including 1 with a linear scar and 2 with hypertrophic scars. A total of 123 postoperative complications (13.2%) were documented. These included wound dehiscence (n = 3), hematoma (n = 3), seroma (n = 1), SSI (n = 46), delayed wound healing at the recipient site (n = 5), and delayed wound healing at the donor site (n = 1). Graft-related complications included partial graft loss in 65 patients and total graft loss in 34 patients. Reoperation was required in 66 patients (7.1%). Patients had an average hospital stay of 8.1 days, with a mean postoperative recovery time of 2.3 months. Average follow-up was 64.5 months, with recurrence typically occurring around 10.0 months after surgery.

### Skin Flaps

A total of 1,193 skin flap procedures were included in the study. Of these, 12 patients received NPWT/VAC. Restricted ROM was reported in 6 patients (0.5%), and 3 patients (0.3%) were dissatisfied with their cosmetic outcome. Disease recurrence occurred in 133 flaps (11.2%), with 67 cases requiring surgical management for recurrence. Scarring was reported in 21 patients, including 20 with linear scars and 1 with a hypertrophic scar. There were 299 documented postoperative complications (25.1%). The most common complication was wound dehiscence (n = 141), followed by SSI (n = 27), delayed wound healing at the recipient site (n = 9), hematoma (n = 8), seroma (n = 2), and delayed wound healing at the donor site (n = 1). Flap-related complications included partial flap loss in 24 patients and total flap loss in 4 patients. Reoperation was required in 68 patients (5.7%). The average hospital stay was 4.7 days, and recovery was relatively quick at 1.4 months. Mean follow-up lasted 21.6 months, and the average time to recurrence was 9.1 months.

## Discussion

### Wide Excision

Wide excision was defined as deroofing followed by healing by secondary intention. This technique remains a cornerstone for managing extensive Hurley Stage II and III disease, typically after acute inflammation has been controlled with antibiotics or incision and drainage.^11^ In our cohort, wide excision had a recurrence rate of 17.2%, aligning with a recurrence rate of 22% reported by Ovadja et al.^9^ While recurrence is not uncommon, this approach allows for radical tissue removal, possibly extending the time to recurrence and lengthening the asymptomatic period.^12^ This is particularly important in complex fistulizing disease, where wide excision may reduce local and distant flares, as well as long-term antibiotic dependence.^13^ However, complications were noted in 7.3% of cases, including wound dehiscence, SSIs, and delayed wound healing, with reoperation required in 1.5%. This group also reported the highest rate of cosmetic dissatisfaction (4.7%) and the second-highest rate of restricted ROM. This is consistent with a recent meta-analysis reporting a 10.6% complication rate following wide excision.^14^ These findings underscore the need to weigh the benefits of disease control against postoperative recovery and cosmetic outcomes, highlighting the need for careful patient selection and standardized wound care.

### Sequential Excision

Sequential excision, defined as staged removal of diseased tissue and often reconstruction,^15^ was the least reported intervention (n = 32). It showed a 28.1% recurrence rate, one postoperative complication, and no reoperations with no reports of cosmetic dissatisfaction or restricted ROM. Despite higher recurrence rates than wide excision or laser therapy, it may offer a function-preserving option in anatomically sensitive or high-risk areas.^16^ Pierazzi et al. highlight the value of sequential excision in managing combined axillary and inguinal disease where large, complex flaps are required and the risk of postoperative complications is heightened.^17^ However, some studies favour a one-staged approach with STSG for cost-effectiveness.^18^ While this technique may be well-suited for patients requiring an individualized surgical plan, clear timelines and expectations for disease management should be established.^15,16^

### Laser Surgery

Laser-assisted surgery demonstrated the second lowest complication (2.2%) and recurrence (5.7%) rates, making it a favourable option for safety and disease control. Immediate hemostasis ensures a clear surgical field, allowing precise sinus tract removal while sparing healthy tissue.^7^ Laser vaporization, under local or general anesthesia, is effective for uncovering deep sinus tissue and managing recurrent, scarred lesions.^7^ Cosmetic dissatisfaction was rare (one case) with no restricted ROM. There were 52 cases of scarring, including hypertrophic scarring in 39 patients. Despite low recurrence rates, some studies suggest earlier recurrence with laser therapy alone, potentially reflecting limitations in treating deeply involved disease. Overall, laser surgery is an effective, minimally invasive intervention best suited for localized or early-stage advanced HS.^19^

### Primary Closure

Primary closure was defined as suturing the wound edges closed following disease excision. While commonly use, it was associated with the highest recurrence (38.0%) and reoperation (9.6%) rates. The intervention’s appeal lies in avoiding skin grafting and complex flaps, simplifying wound management for non–plastic surgeons.^20,21^ However, closure site tension, especially with inadequate margins, likely contributes to recurrence.^22^ A meta-analysis by Cucu et al. similarly reported a higher recurrence rate (25%) with this intervention.^23^ Primary closure also carried the highest complication rate (29.4%) among all modalities, mainly due to wound dehiscence (n = 120), delayed wound healing, and SSI. Cosmetic dissatisfaction and restricted ROM were common. These findings suggest that while primary closure may expedite short-term healing, it is associated with long-term burden in severe HS where tension-free closure is difficult to achieve.^22^ Thus, it should be considered in cases of mild-to-moderate HS.^24^

### Grafts

Skin grafting offers a practical and widely used approach for resurfacing large surgical defects. Grafts were associated with a lower complication rate than flaps (13.2% vs. 25.1%); however, they introduced unique technical challenges, including graft loss and SSIs. Graft failure may result from infection, hematoma, seroma, hypergranulation, and impaired wound healing—often worsened by the chronic inflammatory nature of HS.^25-27^ Notably, only 26.2% of grafts in our cohort used NPWT, a strategy shown to improve graft take by enhancing wound bed vascularity, reducing bacterial load, and promoting granulation tissue formation.^28^ Incorporating NPWT into STSG wound care is important to consider in reconstructive planning. Although grafting accelerates wound coverage compared with healing by secondary intention, we observed a 9.0% recurrence rate, similar to the 6.0% rate reported by Mehdizadeh et al.^29^ STSG may reduce recurrence risk by removing pilosebaceous units, a driver of HS pathogenesis.^30^ However, drawbacks to this intervention include donor site morbidity, suboptimal aesthetic outcomes, and functional impairments.^31^

### Flaps

Skin flaps offer a robust and durable reconstructive option for extensive or recurrent HS.^32^ Unlike grafts, flaps retain their own blood supply, supporting wound healing, tissue resilience, and a reduced risk of complications.^33^ Our cohort showed a 25.1% complication rate, likely reflecting patient selection bias, as flaps are often reserved for severe and anatomically challenging regions where primary closure or grafting is not feasible.^34^ Despite the increased technical demands of flap surgery and the potential for complications such as flap necrosis, wound dehiscence, or contour deformities, flaps offer tension-free closure of large defects and radical excision of diseased tissue.^35^ Several flap types are used in reconstruction, including local advancement, perforator, and regional musculocutaneous flaps. The thoracodorsal artery perforator (TDAP) flap has shown favourable results in axillary reconstruction, offering a thin, pliable tissue coverage and minimal donor site morbidity.^36,37^

Wormald et al. found that TDAP flaps yielded fewer complications, faster recovery, and better quality of life than STSGs.^38^ Similarly, Simone et al., reported that patients receiving TDAP flaps had significantly faster recovery, as well as fewer complications and overall number of procedures, than those undergoing wide excision. These findings support flap surgery as a valuable option in select patients requiring extensive reconstruction.

### Limitations

While this systematic review provides important insights into HS surgical outcomes, several limitations exist. Outcomes were not stratified by intraoperative variables (e.g., margin size, depth of excision, and operative tools). Additionally, follow-up durations varied across studies, limiting and long-term efficacy comparisons. Minor complications may have been underreported, particularly in retrospective and outpatient settings. Furthermore, important clinical factors (e.g., comorbidities, lesion location) were inconsistently reported. Postoperative care details, including wound management and antibiotic use, were infrequently described and may have affected complication rates. Importantly, no matching was performed for disease severity or lesion count, limiting comparisons across surgical modalities given the heterogeneity between Hurley Stage II and III patients.

### Future Directions

Future studies should emphasize individualized surgical planning tailored to lesion location, disease severity, and patient-specific risk factors. Long-term prospective studies with standardized follow-up are needed to compare recurrence and complication rates across interventions. Greater inclusion of patient-reported outcomes, such as DLQI, pain, and outcome satisfaction, is essential. Trials exploring combined surgical and biologic interventions may further inform multidisciplinary management of advanced HS.

## Conclusion

Surgical outcomes in HS differ markedly by technique. Primary closure had the highest rates of postoperative complications, recurrence, and reoperation, despite its technical ease. Skin flaps also showed high complication rates, although recurrence and reoperation rates were lower.

Grafts, often used with NPWT/VAC, carried risks of graft loss, but offered lower recurrence. Laser surgery demonstrated the fewest complications, while sequential excision showed minimal adverse outcomes. Wide excision offered balanced results with moderate complication and recurrence rates, but the highest cosmetic dissatisfaction. Restricted ROM was most common following primary closure and grafting. These findings underscore the need for individualized surgical planning for the management of HS based on disease severity, anatomical site, and patient-centred goals for recovery, function, and aesthetics.

## Supplemental Material

sj-docx-1-cms-10.1177_12034754251391811 – Supplemental material for Surgical Interventions in Advanced Hidradenitis Suppurativa: A Systematic Review Supplemental material, sj-docx-1-cms-10.1177_12034754251391811 for Surgical Interventions in Advanced Hidradenitis Suppurativa: A Systematic Review by Michal Moshkovich, Edgar Akuffo-Addo, Emily Volfson, Jonah Perlmutter, Rebecca Yakubov, Romy Levy, Amrit Thandi, Ammar Saed Aldien, Katya Peri and Ilya Mukovozov in Journal of Cutaneous Medicine and Surgery

sj-docx-2-cms-10.1177_12034754251391811 – Supplemental material for Surgical Interventions in Advanced Hidradenitis Suppurativa: A Systematic Review Supplemental material, sj-docx-2-cms-10.1177_12034754251391811 for Surgical Interventions in Advanced Hidradenitis Suppurativa: A Systematic Review by Michal Moshkovich, Edgar Akuffo-Addo, Emily Volfson, Jonah Perlmutter, Rebecca Yakubov, Romy Levy, Amrit Thandi, Ammar Saed Aldien, Katya Peri and Ilya Mukovozov in Journal of Cutaneous Medicine and Surgery

sj-docx-3-cms-10.1177_12034754251391811 – Supplemental material for Surgical Interventions in Advanced Hidradenitis Suppurativa: A Systematic Review Supplemental material, sj-docx-3-cms-10.1177_12034754251391811 for Surgical Interventions in Advanced Hidradenitis Suppurativa: A Systematic Review by Michal Moshkovich, Edgar Akuffo-Addo, Emily Volfson, Jonah Perlmutter, Rebecca Yakubov, Romy Levy, Amrit Thandi, Ammar Saed Aldien, Katya Peri and Ilya Mukovozov in Journal of Cutaneous Medicine and Surgery

sj-docx-4-cms-10.1177_12034754251391811 – Supplemental material for Surgical Interventions in Advanced Hidradenitis Suppurativa: A Systematic Review Supplemental material, sj-docx-4-cms-10.1177_12034754251391811 for Surgical Interventions in Advanced Hidradenitis Suppurativa: A Systematic Review by Michal Moshkovich, Edgar Akuffo-Addo, Emily Volfson, Jonah Perlmutter, Rebecca Yakubov, Romy Levy, Amrit Thandi, Ammar Saed Aldien, Katya Peri and Ilya Mukovozov in Journal of Cutaneous Medicine and Surgery
