# Supplementary material for: Surgical Interventions in Advanced Hidradenitis Suppurativa: A Systematic Review
Source: J Cutan Med Surg. 2025 Nov 12;30(3):282–8. doi: 10.1177/12034754251391811 (PMC13216569; doi:10.1177/12034754251391811)
Supplement: sj-docx-1-cms-10.1177_12034754251391811 – Supplemental material for Surgical Interventions in Advanced Hidradenitis Suppurativa: A Systematic Review [file sj-docx-1-cms-10.1177_12034754251391811.docx]

**Supplemental Table 1.** Search strategy for literature screening.

Ovid MEDLINE and Embase <1970 to 2024 October 17>

| **#** | **Search Terms** | | **Results** |
| --- | --- | --- | --- |
| 1 | Disease | "Suppurative hidradenitis" or "Acne inversa" or "apocrinitis" or "hidradenitis suppurativa" or "suppurativa hidradenitis" or "Verneuil’s disease" | 9088 |
| 2 | Treatment | "wide excision" or "excision" or "wide local excision" or "wide local resection" or "wide resection" or "deroofing" or "deroofing surgery" or "laser surgery" or "carbon dioxide laser" or "CO2 laser surgery" or "neodymium YAG laser" | 145,447 |
| 3 | Final Search | 1 and 2 | 647 |
