# Supplementary material for: Surgical Interventions in Advanced Hidradenitis Suppurativa: A Systematic Review
Source: J Cutan Med Surg. 2025 Nov 12;30(3):282–8. doi: 10.1177/12034754251391811 (PMC13216569; doi:10.1177/12034754251391811)
Supplement: sj-docx-2-cms-10.1177_12034754251391811 – Supplemental material for Surgical Interventions in Advanced Hidradenitis Suppurativa: A Systematic Review [file sj-docx-2-cms-10.1177_12034754251391811.docx]

**Supplemental Table 2.** Flow diagram of literature screening using the Preferred Reporting Items for Systematic Reviews and Meta-Analyses (PRISMA) guidelines. Figure adapted from http://prisma-statement.org.

**
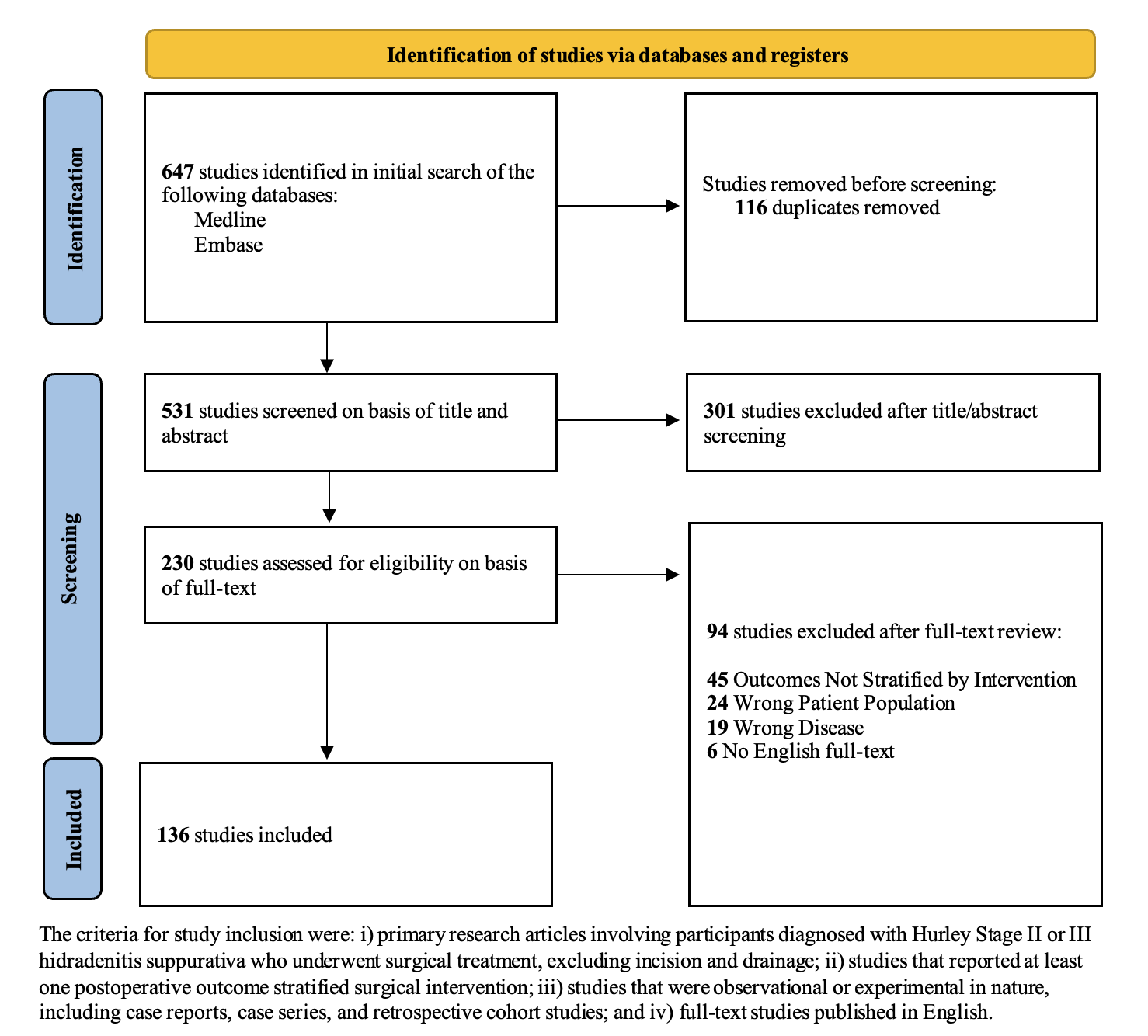
**
