# Supplementary material for: Surgical Interventions in Advanced Hidradenitis Suppurativa: A Systematic Review
Source: J Cutan Med Surg. 2025 Nov 12;30(3):282–8. doi: 10.1177/12034754251391811 (PMC13216569; doi:10.1177/12034754251391811)
Supplement: sj-docx-3-cms-10.1177_12034754251391811 – Supplemental material for Surgical Interventions in Advanced Hidradenitis Suppurativa: A Systematic Review [file sj-docx-3-cms-10.1177_12034754251391811.docx]

**Supplemental Table 3.** Summary demographics and disease characteristics of hidradenitis suppurativa patient cohort.

| **BASELINE CHARACTERISTICS** | **n (%)** |
| --- | --- |
| Number of Participants | 3419 |
| Mean Age (Years) | 35.5 |
| Sex |  |
| Female | 1770 (52) |
| Male | 1224 (36) |
| Unknown | 425 (12) |
| BMI (kg/m²) | 30.5 |
| **Race/Ethnicity** |  |
| Caucasian & White | 225 (7) |
| Asian | 18 (0.5) |
| Hispanic | 7 (0.2) |
| Black & African American | 261 (8) |
| Unknown or Other | 2908 (85) |
| **Comorbidities** |  |
| Smoking | 1117 (33) |
| Diabetes | 171 (5) |
| Hypertension | 139 (4) |
| Acne | 120 (4) |
| Inflammatory Bowel Disease | 46 (1) |
| Polycystic Ovary Syndrome | 18 (0.5) |
| Depression | 12 (0.4) |
| **Disease Location** |  |
| Axilla | 2200 (39) |
| Genital | 1802 (32) |
| Perianal | 773 (14) |
| Mammary | 257 (5) |
| Other | 355 (6) |
| Not Reported | 259 (5) |
| **Disease Staging** |  |
| Hurley Stage II | 569 (17) |
| Hurley Stage III | 871 (25) |
| > Stage 2 but Indeterminate | 1979 (58) |
| **Previous HS Therapy** |  |
| Systemic Antibiotics | 1192 (35) |
| Biologics | 294 (9) |
| Incision & Drainage | 196 (6) |
| Hormonal Therapies | 138 (4) |
| Steroids | 90 (3) |
| **Surgical Intervention Received (Number of Procedures)** |  |
| Wide Excision | 1923 (34) |
| Flap | 1193 (21) |
| Surgical Excision w/ Primary Closure | 1112 (20) |
| Skin Graft - Split Thickness | 715 (13) |
| Skin Graft - Other | 218 (4) |
| Laser Surgery - Carbon Dioxide (CO2) | 333 (6) |
| Laser Surgery - Other | 120 (2) |
| Sequential Partial Excision | 32 (0.6) |
