# Supplementary material for: Surgical Interventions in Advanced Hidradenitis Suppurativa: A Systematic Review
Source: J Cutan Med Surg. 2025 Nov 12;30(3):282–8. doi: 10.1177/12034754251391811 (PMC13216569; doi:10.1177/12034754251391811)
Supplement: sj-docx-4-cms-10.1177_12034754251391811 – Supplemental material for Surgical Interventions in Advanced Hidradenitis Suppurativa: A Systematic Review [file sj-docx-4-cms-10.1177_12034754251391811.docx]

**Supplemental Table 4.** Hidradenitis suppurativa postoperative outcomes stratified by surgical intervention.

| **Recovery Outcomes** | **Wide Excision**  **(n = 1923)** | **Sequential Excision**  **(n = 32)** | **Laser**  **(n = 453)** | **Primary Closure**  **(n = 1112)** | **Graft**  **(n = 933)** | **Flap**  **(n = 1193)** |
| --- | --- | --- | --- | --- | --- | --- |
| Negative Pressure Wound Therapy (NPWT)/Vacuum-assisted Closure (VAC) | 135 (7) | 0 (0) | 12 (2.6) | 15 (1.3) | 218 (23.4) | 12 (1) |
| **Total Complications** | 140 | 1 | 10 | 327 | 123 | 299 |
| Dehiscence (n) | 12 | 1 | 2 | 120 | 3 | 141 |
| Hematoma (n) | 1 | 0 | 0 | 2 | 3 | 8 |
| Seroma (n) | 0 | 0 | 0 | 5 | 1 | 2 |
| Surgical Site Infection (n) | 19 | 0 | 3 | 41 | 46 | 27 |
| Delayed Wound Healing at  Recipient Site (n) | 8 | 0 | 0 | 66 | 5 | 9 |
| Delayed Wound Healing at  Donor Site (n) | 0 | 0 | 0 | 0 | 1 | 1 |
| Partial Graft Loss (n) |  |  |  |  | 65 |  |
| Total Graft Loss (n) |  |  |  |  | 34 |  |
| Partial Flap Loss (n) |  |  |  |  |  | 24 |
| Total Flap Loss (n) |  |  |  |  |  | 4 |
| Hypertrophic Scar (n) | 0 | 0 | 39 | 2 | 2 | 1 |
| Linear Scar (n) | 0 | 0 | 13 | 9 | 1 | 20 |
| Average Recovery Time (Months) | 2.8 (n = 919) | 2.0  (n = 32) | 2.3  (n = 236) | 2.0  (n = 519) | 2.3  (n = 122) | 1.4  (n = 190) |
| Participants with Restricted Range of Motion (n) | 24 | 0 | 0 | 30 | 23 | 6 |
| Participants Dissatisfied with Cosmetic Outcome (n) | 90 | 0 | 1 | 42 | 27 | 3 |
| Re-operation (n, %) | 29 (1.5) | 0 (0) | 3 (0.7) | 107 (9.6) | 66 (7.1) | 68 (5.7) |
| Disease Recurrence (n, %) | 331 (17.2) | 9 (28.1) | 26 (5.7) | 423 (38) | 84 (9) | 133 (11.2) |
| Average Time Elapsed Since Surgery for Recurrence (Months) | 7.3  (n = 275) | NR | 6.5  (n = 46) | 9.3  (n = 107) | 10.0  (n = 91) | 9.1  (n = 138) |
| Surgery for Recurrence (n, %) | 24 | 0 (0) | 1 | 124 | 39 | 67 |
| Average Hospital Stay Duration (Days) | 7.3 (n = 441) | NR | 0.5  (n = 25) | 2.8  (n = 176) | 8.1  (n = 287) | 4.7  (n = 515) |
| Average Follow-up Duration (Months) | 36.2  (n = 1689) | 20.4  (n = 14) | 35.2  (n = 291) | 37.9  (n = 279) | 64.5  (n = 630) | 21.6  (n = 766) |
